# Monocarboxylate Transporter 1 May Benefit Cerebral Ischemia *via* Facilitating Lactate Transport From Glial Cells to Neurons

**DOI:** 10.3389/fneur.2022.781063

**Published:** 2022-04-25

**Authors:** Mao Zhang, Yanyan Wang, Yun Bai, Limeng Dai, Hong Guo

**Affiliations:** Department of Medical Genetics, College of Basic Medical Sciences, Army Medical University, Chongqing, China

**Keywords:** monocarboxylate transporter 1 (MCT1), lactate transport, cerebral ischemia, astrocytes, oligodendrocytes (OLs)

## Abstract

Monocarboxylate transporter 1 (MCT1) is expressed in glial cells and some populations of neurons. MCT1 facilitates astrocytes or oligodendrocytes (OLs) in the energy supplement of neurons, which is crucial for maintaining the neuronal activity and axonal function. It is suggested that MCT1 upregulation in cerebral ischemia is protective to ischemia/reperfusion (I/R) injury. Otherwise, its underlying mechanism has not been clearly discussed. In this review, it provides a novel insight that MCT1 may protect brain from I/R injury *via* facilitating lactate transport from glial cells (such as, astrocytes and OLs) to neurons. It extensively discusses (1) the structure and localization of MCT1; (2) the regulation of MCT1 in lactate transport among astrocytes, OLs, and neurons; and (3) the regulation of MCT1 in the cellular response of lactate accumulation under ischemic attack. At last, this review concludes that MCT1, in cerebral ischemia, may improve lactate transport from glial cells to neurons, which subsequently alleviates cellular damage induced by lactate accumulation (mostly in glial cells), and meets the energy metabolism of neurons.

## Introduction

Cerebral ischemia is a worldwide public health issue that causes brain dysfunction and most frequently results in mortality (1). Ischemia/reperfusion (I/R) injury is the underlying pathogenesis of neurological damage in ischemic stroke (2). Intravenous injection of lactate offers neuroprotection at the reperfusion period of cerebral ischemia (3). Administration of lactate reduces brain lesion volume, ameliorates behavioral outcomes, and promotes long-term memory in neonatal hypoxia-ischemia (4, 5). These pieces of evidence point out that lactate plays a beneficial role in cerebral ischemia. In the work of Sylvain et al. (6) using a photothrombotic stroke model, they have shown a link between brain energy metabolism and increased glycogen level. Actually, lactate is not only an energetic source of neurons, but also acts as a signaling molecule in the regulation of neuronal excitotoxicity (7–9).

In the central nervous system (CNS), lactate is mainly produced by astrocytes and oligodendrocytes (OLs), and then metabolized in neurons to sustain neuronal growth (10, 11). In addition, Kitchen et al. (12) work demonstrates that the pharmacological inhibition of protein aquaporin-4 expressed in astrocytes prevents the development of cerebral edema and promotes functional recovery in injured rats. Therefore, the regulation of astrocytes and OLs in relation with neuronal injury, especially in the aspect of lactate transport, can benefit cerebral ischemia.

Monocarboxylate transporter 1 (MCT1) is an effective and the most abundant lactate transporter in the brain (11), which is expressed in OLs, astrocytes, and some population of neurons (13). Importantly, MCT1 promotes lactate transport from astrocytes and OLs to neurons (14). Moreover, neuronal MCT1 upregulation has a protective effect on cerebral I/R injury (15). Besides, the upregulation of astrocytic MCT1 expression improves neurological deficit in middle cerebral artery occlusion rats (16). MCT1, located in the inner mitochondrial membrane of astrocytes, facilitates lactate entry into tricarboxylic acid (TCA) cycle (17). Thus, MCT1 in the regulation of lactate transport from astrocytes and OLs to neurons can benefit cerebral ischemia.

In this work, it suggests that MCT1 can benefit cerebral ischemia *via* regulating lactate flow from glial cells to neurons. Specifically, this review introduces the structure and function of MCT family, and then discusses the regulation of MCT1 in lactate transport between glial cells, such as astrocytes, OLs, and neurons. Finally, it proposes that MCT1 benefits neuronal damage in cerebral ischemia. Besides, lactate efflux from astrocytes and OLs attenuates accumulated lactate-induced cellular response in these cells. Hence, this review concludes that the regulation of MCT1 in lactate balance among neural cells is protective to the neuronal I/R injury that should be a novel therapeutic target for cerebral ischemia.

## Structure and Distribution of Monocarboxylate Transporter Family

**Figure 1:**
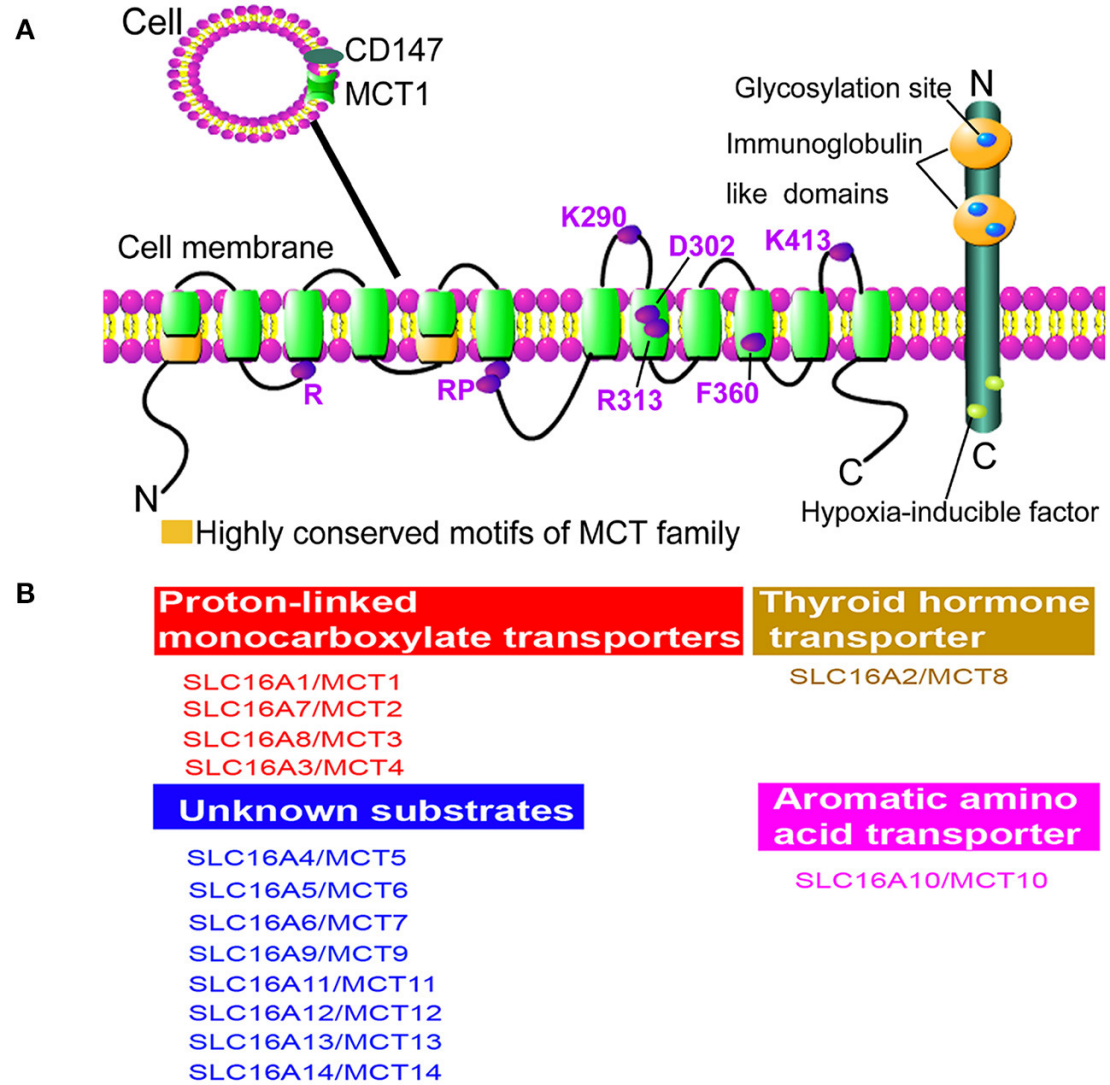
Structure and function of monocarboxylate transporter (MCT) family. **(A)** The proposed topology of MCT family. There are 10–12 α-helical transmembrane domains (TMDs) for MCT family membranes in the presence of N- and C-termini located within cytoplasm. Besides, it shows the greatest variation at N- and C-termini and the large loop between TMDs 6 and 7 among family members, while the TMDs themselves are highly conserved. Importantly, two highly conserved motifs can be identified as the characteristic of the MCT family (green). Cluster of differentiation 147 (CD147) is an ancillary protein of MCT1 and MCT4, which is a cell surface glycoprotein containing a signal transmembrane span, two immunoglobulin-like domains in the extracellular region, and a short C-terminal cytoplasmic tail. The marked amino acids are significant for the structure conservation of MCT family. Specifically, R represents Arg; RP represents Arg and Pro; K290 represents Lys290; D302 represents Asp302; R313 represents Arg313; F360 represents Phe360; K413 represents Lys413; E218 represents Glu218. **(B)** Functions of MCT family members. MCT1, MCT2, MCT3, and MCT4 are proton-dependent transporters, which transport lactate, pyruvate, D-β-hydroxybutyrate, and acetoacetate (1). MCT8 can transport thyroid hormone and MCT10 is a transporter of aromatic amino acids, while the substrates of other MCT members are still unknown.

Human SLC16 gene family, also known as MCT family, comprises 14 members (MCT1–MCT14) (13). The molecular identity of the first member of this family is MCT1, which was established by two parallel studies: specific labeling studies in erythrocytes of rats and rabbits (18); studies in the purification and N-termini sequencing of a 40- to 50-kDa protein (19). Besides, the structures of other MCT members were further identified, showing similar topography. Normally, MCT family has 10–12 α-helical transmembrane domains (TMDs) with intracellular N- and C-termini and a large intracellular loop between TMDs 6 and 7, already confirmed for MCT1 in erythrocytes (18). Especially, the cluster of differentiation 147 (CD147) is required by MCT1 and MCT4 (20). The proposed topology of MCT family is schemed (Figure 1A). Experimentally, it is demonstrated that MCT1–MCT4 catalyzes the proton-linked transport of metabolically important monocarboxylates, such as lactate, pyruvate, and ketone bodies. MCT8 has a high affinity for thyroid hormone, and MCT10 is a transporter of aromatic amino acid (21, 22). In addition, substrates transported by the other eight members (MCT5, MCT6, MCT7, MCT9, MCT11–MCT14) remain unknown (22). In non-mammalian, five members (MCT1–MCT5) in yeast are not responsible for lactate transport (23) (Figure 1B).

## MCT1 is Expressed in Glial Cells and Neurons

The conceptual role of lactate in the brain has shifted from glycolytic waste product to supplemental fuel or signaling molecule (24). Four members of MCT family (MCT1, MCT2, MCT3, and MCT4) facilitate cerebral lactate transport, while the cerebral distribution of them is quite different. According to the brain-RNAseq database (http://www.brainrnaseq.org/), MCT1 is predominantly expressed by endothelial cells, MCT2 is equally expressed by all major brain cell types, and MCT3 and MCT4 are mainly expressed by microglia. Particularly, MCT1 is recognized as a lactate transporter of erythrocyte, and then found in energy-costing tissues, such as Chinese hamster ovary, Xenopus laevis oocyte and brain (25, 26). In the brain, MCT1 is distributed in the most of neural cells, such as astrocytes, OLs, and neurons (26). In addition, MCT1 expression can be merely recognized in activated microglia, which is merely correlated with inflammation (27). It is identified that MCT1 mainly localizes to OLs, astrocytes, and some specific neuronal populations, such as hippocampal, brainstem trigeminal ganglion, and cerebellar Purkinje neurons (11, 28, 29) (Table 1).

**Table 1:** Expression and Distribution of MCT1.

**Distribution**	**Time**
**MCT1 Expression**	
Erythrocyte, Endothelial cell	1992 (17)
Chinese hamster ovary	1994 (26)
Xenopus laevis oocyte	1998 (27)
Astrocyte, Oligodendrocyte, Microglia, Neuron	1999 (28, 29)
Oligodendrocyte, Astrocyte, Some population of neuron	2012 (16, 29, 30)

In cerebral ischemia, an increased expression of MCT1 is protective to neuronal damage (2, 4). Redistribution of MCT1 also protects OLs from ischemic stress (29). Brain cost 10 times higher than what should be expected from its weight alone (30). Lactate, transported by MCT1, is an indispensable energy source of neurons (31). Moreover, the developmental expression of oligodendrocyte MCT1 has a regulative effect on neuronal amounts in medial prefrontal cortex (mPFC) during 12 months (32). Thus, MCT1 protects neurons from I/R injury through mediating lactate balance among neural cells.

## MCT1 Couples Astrocyte-to-Neuron Lactate Flow

Astrocytes exhibit a high glycolytic rate and release large amounts of lactate to neurons (11). In 1994, Pellerin and Magistretti has proposed a hypothesis of astrocyte-neuron lactate shuttle (ANLS). In ANLS, astrocytes serve as a “lactate source” whereas neurons serve as a “lactate sink” (33). Moreover, the opposition by Bak et al. (34) who argued that the oxidative metabolism of lactate in neurons only occurs during repolarization (and in the period between depolarization) rather than during neurotransmission activity. Hence, it still exists a debate in ANLS hypothesis. Until recently, it is discussed that the lactate supplement of astrocytes is a necessary energetic source of neurons (35). Furthermore, the glycolytic capability of astrocytes can be stimulated under various conditions, such as hypoxia, acidosis, and ATP deficiency (36). As an example, glutamate, released from activated neurons, is considered as a stimulus of glycolysis in astrocytes (37).

Lactate release is enhanced during glycogenolysis and glycolysis (38). A glucose flow exists between astrocytes and neurons. Glucose passes through blood brain barrier (BBB), and then transported and stored in a form of glycogen in astrocytes (39, 40). Glycogen is not only a component of brain energetics during sensory stimulation (41, 42), but also a regulator of K^+^ inflow, glutamate uptake, and calcium homeostasis (43, 44). Particularly, glycogen can be rapidly converted to pyruvate/lactate, or used for glutamate biosynthesis and glucose production (45, 46). Either glucose or lactate can be exported from astrocytes to neurons and used as an energetic substrate (47). Otherwise, under ischemia or hypoxia condition, lactate becomes the main energetic source of neurons (48). At this moment, lactate is converted from glycogen in astrocytes, and transported into neurons (49). In an experiment *in vitro*, astrocytic lactate release is stimulated by glutamate (50).

In the CNS, MCT1 arouses much attention because of its unique localization, which is expressed in astrocytes and neurons (11). Meanwhile, MCT2 restrictively localizes to neurons (7) and MCT4 is mainly expressed by astrocytes (31). Such a cellular distribution of cerebral MCT1, MCT2, and MCT4 may associate with their functional characteristics. *In vitro*, it was observed in cultured tanycytes that MCT1 is functional for lactate influx, displaying a Km of 7.7 mM (51, 52) and MCT2 has a Km of 0.8 mM for lactate influx (53), while MCT4 displays a Km of 34 mM for lactate efflux (54). Notably, MCT1 can also export lactate from astrocytes into extracellular space (55). Actually, both MCT1 and MCT4 can convey astrocyte-released lactate, while MCT1 is involved in basal lactate release, and MCT4 is required for enhanced lactate export (56–58). MCT2, predominantly expressed in neurons (59), mainly regulates the lactate uptake of neurons whose activity can be stimulated by glutamate (60).

**Figure 2:**
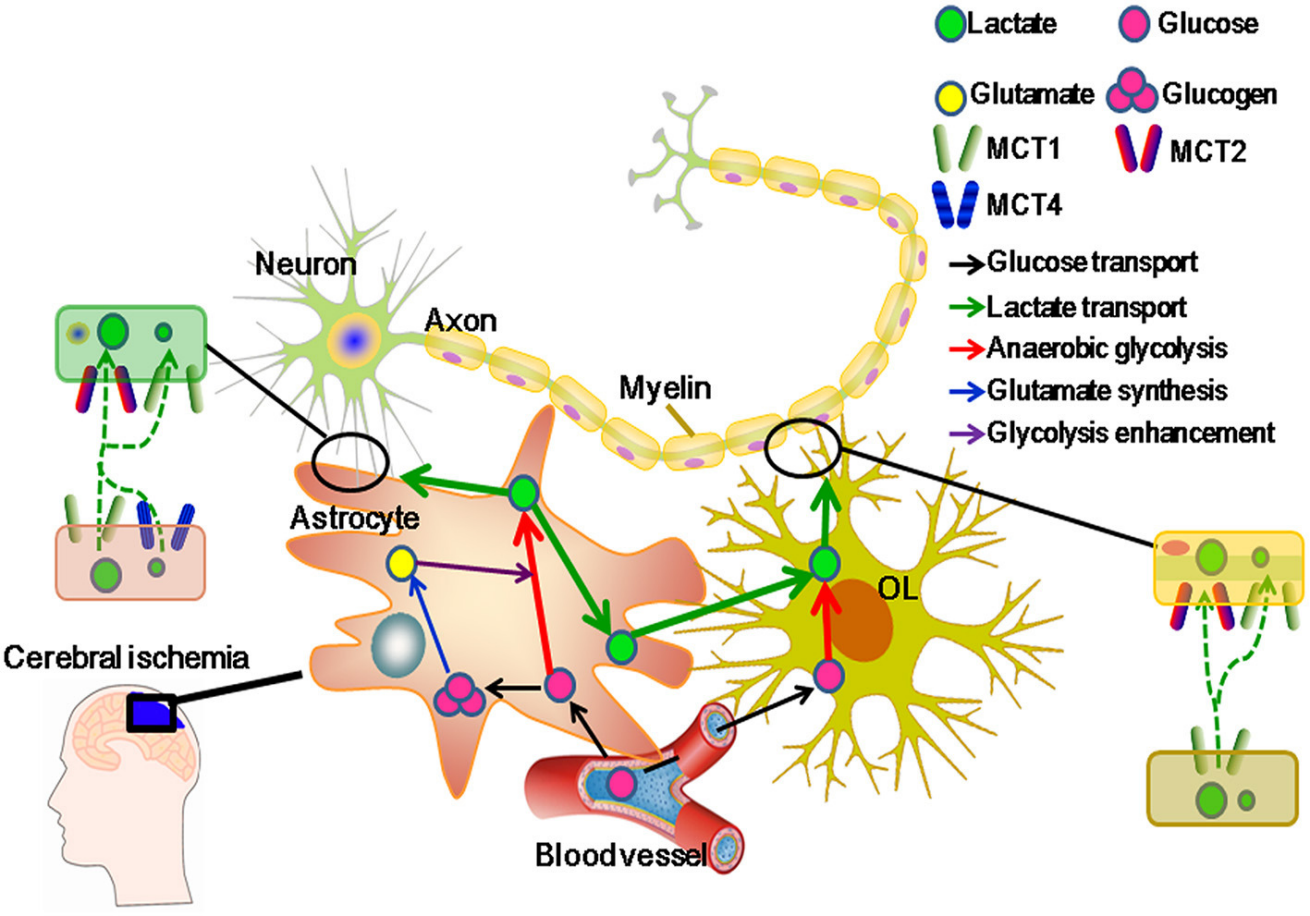
Monocarboxylate transporter 1 (MCT1) regulatively facilitates lactate transport from glial cells to neurons in cerebral ischemia. MCT1 is expressed in glial cells, such as astrocytes and oligodendrocytes (OLs), and some populations of neurons in the areas of hippocampus, brainstem, trigeminal ganglion, and cerebellar Purkinje. Meanwhile, another three lactate transporters (MCT2, MCT3, and MCT4) are restricted to cell types: MCT2 in neurons, MCT3 in epithelial cells, and MCT4 in astrocytes. Moreover, MCT1 can regulate lactate influx and efflux among neural cells. MCT2 can merely regulate lactate influx and MCT4 mainly functions in lactate efflux from neural cells. In cerebral ischemia, MCT1 upregulation protects neurons from I/R injury through regulating lactate transport (2). Generally, glucose is transported from capillaries to glial cells, and then to be polymerized into glucogen (stored in astrocytes) or aerobically metabolized. Otherwise, increased glutamate is released from neurons and activates glial glycolysis in cerebral ischemia. As a result, glucose is metabolized into lactate in glial cells. At this moment, lactate becomes the main energetic substrate of neurons. To adapt the changed microenvironment, e.g., increased acidosis, MCT1 activity is strengthened, which not only facilitates the accumulated lactate efflux from glial cells, but also improves the lactate influx to neurons (11).

Here, it is suggested that glucose is crossed over BBB, and then polymerized and stored in astrocytes in a form of glycogen. Once energetic supplements are rapidly required for neurons, e.g., under ischemic attack, astrocytic glycogen is activated and metabolized into lactate and glutamate. Besides, glutamate can further stimulate glycolysis, enhancing astrocytic lactate release. At this moment, MCT1 couples lactate flow from astrocytes to neurons, which exports lactate from astrocytes, and possibly imports lactate into neurons. Moreover, MCT4 can further enhance astrocytic lactate release in the presence of MCT1-mediated lactate efflux, and MCT2 facilitates lactate influx into neurons (Figure 2).

## MCT1 Facilitates Lactate Transport From OLS to Neurons

Myelin wraps up 99% of axons, which is critical for an efficient impulse conduction of axons. Otherwise, myelin limits the contact between axons and extracellular space, and restricts the access of energetic metabolites into axons (61). Besides, it is postulated that axons derive metabolic energy from OLs (62). *In vitro*, oligodendrocyte MCT1 metabolically supports neuronal survival, and its deficiency leads to cell death (11). *In vivo*, OLs and myelin benefit the neurodegeneration, axonal energy metabolism, and structural integrity of axons. In addition, lactate supports oligodendrocyte development and myelination (63). The promotion of MCT1 in lactate transport from OLs to axons encourages neuronal conduction (62, 64).

Monocarboxylate transporter 1 was first found in the endothelial cells of capillaries and astrocytes (62, 65, 66). Until recently, MCT1 expressed in OLs was discussed. It is suggested that lactate is released from OLs through MCT1, and utilized by axons as an energetic substrate (62). Indeed, MCT1 is highly enriched within OLs, whose disruption or downregulation induces axon damage and neuron loss in an animal or a cell culture model (15, 62). In addition, MCT2 is expressed in neurons and MCT4 in astrocytes (67–69). MCT1 is indispensable for glycolytic OLs in supporting neuronal metabolism (70). On one hand, oligodendroglial MCT1 regulates lactate efflux and prevents the intracellular accumulation of lactate in OLs. On the other hand, MCT1 guarantees sufficient lactate to be transported and metabolized in neuronal axons (11). Furthermore, capillaries provide a constant source of glucose which enters astrocytes or OLs, and undergoes glycolysis. Then, glycolytic products (lactate or pyruvate) in OLs diffuse through cytoplasmic (“myelinic”) channels and reach the periaxonal space *via* MCT1 (15, 63). Moreover, MCT1, expressed in astrocytes, especially in the population closely located to OLs, constantly supplies lactate to neurons (71). Above all, MCT1 regulates lactate flow released from OLs to neurons that energetically supports the neuronal activity (Figure 2).

## MCT1 Protects Brain From I/R Injury *via* Regulating Lactate Transport

Glycolysis is significant for neuronal metabolism in cerebral ischemia (72). Glucose enters brain parenchyma *via* glucose transporters, which was partially converted to lactate in astrocytes and OLs to meet the energetic demand of neurons (62, 67). It was proved that the given of exogenous lactate rescues impaired long-term memory (73, 74). In addition, lactate supports electrically evoked action potentials in brain slices when suffering oxygen and glucose deprivation (75). Indeed, lactate oxidation can support cellular functions under specific experimental conditions, e.g., lactate infusions or strenuous exercise (76). Even, lactate may “jump start” neuronal recovery after the restoration of blood flow and oxygen delivery (77). Therefore, lactate is an obligatory energy substrate for neurons under I/R injury (78).

Furthermore, the expressions of MCT1, MCT2, and MCT4 are cell-specifically modulated to adapt the changed metabolic state in cerebral ischemia (12). Particularly, MCT1 expression is strongly enhanced both at 1 and 24 h post-ischemia, which is supposed to regulate the lactate supplement from microvessels to ischemic brain and redistribute lactate between glial cells (astrocytes and OLs) and neurons (15). Besides, it has been recognized that post-ischemic modulation of MCT1 in rat hippocampal CA1 neurons can benefit their survival (79). Lactate is released in larger quantities from “resting” cultured astrocytes than that from neurons despite both of them can produce lactate under various conditions, e.g., high K^+^ or dinitrophenol (an uncoupling agent that suppresses oxidative respiratory chain) exposure (52, 53). Besides, OLs provide lactate to support neuronal survival and axonal energy metabolism (11). Here, it proposes a novel concept that in cerebral ischemia, MCT1 can metabolically support the neuronal survival and activity through regulating lactate transport (80).

In cerebral ischemia, the dysfunction of MCT1 is a critical determinant of acid-related cell damage (16, 81). Otherwise, MCT1 upregulation can alleviate cellular damage, and further improve the neurological deficit following experimental transient focal cerebral ischemia (16). The demonstrated utilization of lactate in hypoxic hippocampal slices suggests that MCT1 can meet the metabolic needs of the injured neurons (82). In ischemic heart, it showed that the increased MCT1 expression is a regulator in restoring cardiac pH through lactate export (83). Similarly, in ischemic brain, enhanced MCT1 immunoreactivity was observed in astrocytes, endothelial cells, and adjacent ependymal lining, which plays a protective role in the initial and long-term neuronal survival in hippocampus (84). Therefore, MCT1 potentially mediates the extent of lactic acidosis and lactate metabolism during cerebral ischemia, through which, neurons can sustain I/R injury.

**Figure 3:**
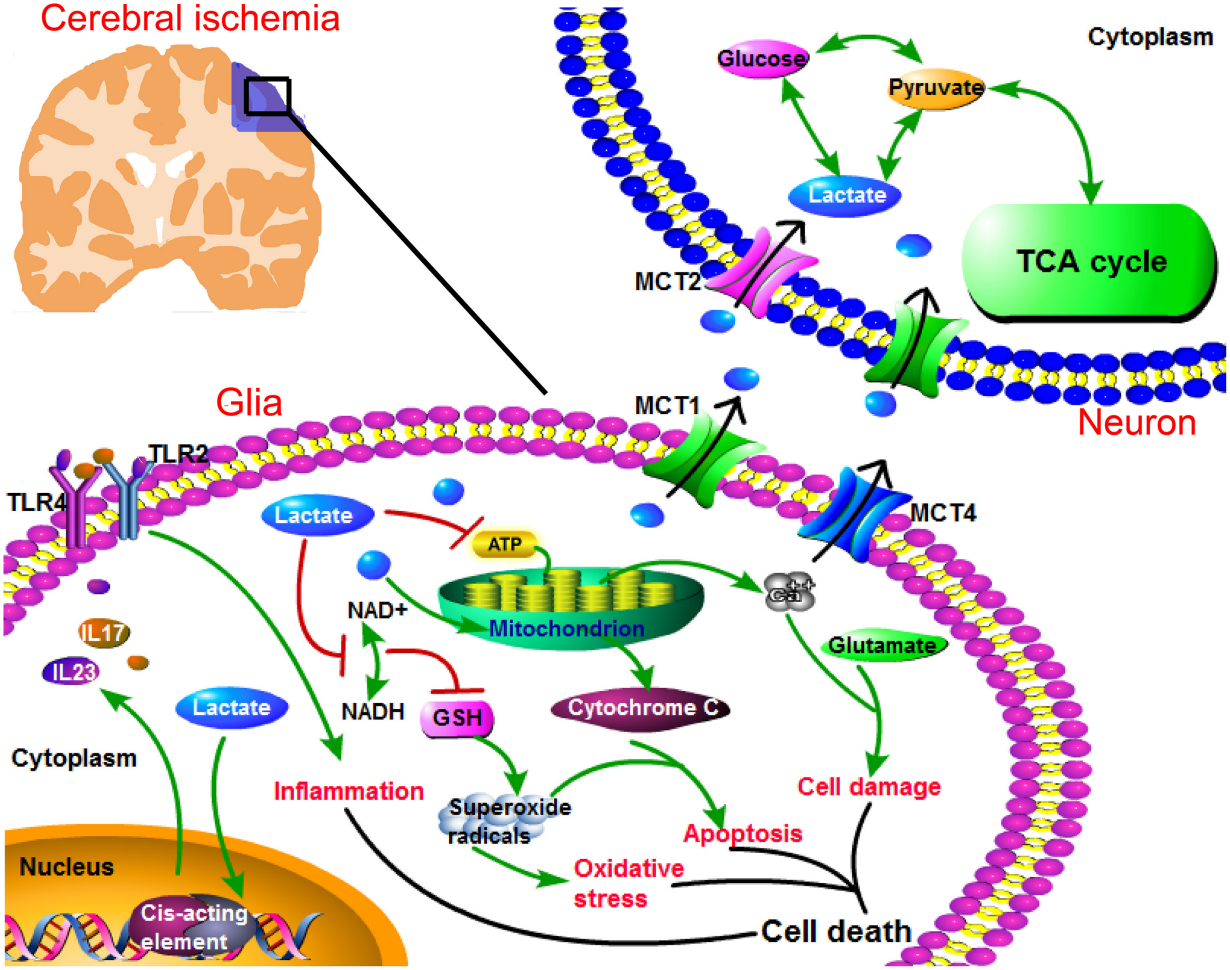
Regulation of MCT1 in lactate transport promotes neuronal energy supplement, and suppresses cellular damage under ischemic attack. In ischemic brain, anaerobic glycolysis is largely enhanced and leads to lactate overproduction. Lactate becomes the main energy resource for neurons, while its intracellular accumulation (in glial cells) leads to toxic response. Specifically, lactate accumulation associates with cellular responses, such as inflammation, oxidative stress, apoptosis, Ca^2+^ overload, and glutamate-induced excitotoxicity. Especially, lactate can promote the transcription of proinflammatory factors (interleukin 23/interleukin 17 (IL23/IL17)), which triggers inflammation *via* toll-like receptor 2/toll-like receptor 4 (TLR2/TLR4). In addition, lactate can suppress the production of glutathione (GSH) which is an indispensable anti-oxidative enzyme, and GSH deficit leads to the increased superoxide radicals. Then, superoxide radicals can further activate oxidative stress. Again, superoxide radicals contribute to apoptosis in the presence of mitochondrial cytochrome C. Furthermore, lactate decreases adenosine triphosphate (ATP) level, resulting in Ca^2+^ overload. Ca^2+^ overload coexists with glutamate-induced excitotoxicity which leads to cellular damage. Collectively, intracellular lactate accumulation contributes to these cellular responses, leading to cell death. Therefore, a timely and rightly utilization of lactate protects brain from I/R injury. MCT1 can facilitate lactate efflux from glial cells, and assist lactate influx into neurons. Besides, neuronal MCT2 assists lactate entries into neurons, and then lactate is efficiently utilized by neurons.

Ischemia/reperfusion injury includes inflammatory reaction, oxidative stress (85, 86), apoptosis (87), and excitotoxicity (88). Overproduced lactate in ischemic brain plays as a mediator to activate proinflammatory pathways, e.g., interleukin 23/interleukin 17 (IL23/IL17) signaling pathway, which further activates toll-like receptor 2/toll-like receptor 4 (TLR2/TLR4) and then induces inflammatory response (89, 90). Additionally, changes in glycolytic intermediates contribute to reductions in the form of nicotinamide adenine dinucleotide (NADH), and glutathione (GSH) (91). Besides, high intracellular lactate level can impede the coupled oxidation of NADH to nicotinamide adenine dinucleotide (NAD^+^) in MCT1 inhibitor-treated cells (92). As a result, GSH reduction facilitates the production of hypoxia-enhanced superoxide radicals and hydrogen peroxide (92, 93). Then, more cytochrome C is released from mitochondria, which further contributes to the cell death through an apoptotic pathway (87). Furthermore, high NADH level favors glycolysis, but not aerobic glycolysis. When glycolysis is enhanced, ATPase activity is suppressed and lactate is overproduced (86). On one hand, the overproduced lactate can exacerbate lactic acidosis. On the other hand, the suppressed activity of ATPase decreases ATP production (87), and promotes an overload of intracellular Ca^2+^, eventually resulting in cell death (87, 88). In addition, the excessive accumulation of intracellular Ca^2+^ coexists with glutamate-induced excitotoxicity and overstimulation N-methyl-D-aspartate type (NMDARs) (94, 95). Both Ca^2+^ overload and excitotoxicity exacerbate cellular damage during cerebral ischemia (Figure 3).

## Conclusions and Perspectives

Glucose is an obligatory fuel of brain, which is metabolized mostly in a manner of aerobic glycolysis, but not anaerobic glycolysis (82). Anaerobic glycolysis and increased lactate production can be extensively enhanced when facing oxygen deficit (92). In glia-neuron metabolic crosstalk, lactate is produced mainly by glial cells, such as astrocytes and OLs, and further utilized by neurons (63). In ANLS model, glutamate depolarizes neurons by its receptors, which is terminated by an efficient glutamate uptake system in astrocytes (96). Especially, glutamate-cotransported Na^+^ can activate Na^+^/K^+^ ATPase, which fuels glycolytic enzymes and stimulates glycolysis in astrocytes. Lactate, once released from astrocytes, can be taken up and utilized by neurons (96). Besides, OLs are critical intermediaries for lactate transport to neurons (97), which potentially impact energy-dependent processes in axons (98). Actually, energetic interactions exist among astrocytes, OLs, and neurons: astrocytes can transfer energetic metabolites directly to neurons, or to OLs which in turn support neuronal axons (47).

Research over the last couple of decades has provided novel insights into lactate neurobiology and the implications of lactate transport-driven neuroenergetics in the health and diseases of peripheral nerve and CNS. Lactate transporters in peripheral nerves are important for the maintenance of axon and myelin integrity, motor end-plate integrity, the development of diabetic peripheral neuropathy (DPN), and the functional recovery following nerve injuries (99). This review discusses that the regulation of MCT1 in ANSL and oligodendrocyte-neuron lactate flow are necessary for the functional recovery of neurons under I/R injury. Especially, MCT1 can peculiarly facilitate astrocytic and oligodendroglial lactate transport to neurons (11). Conversely, the dysfunction of MCT1 may impede lactate transport from glial cells to neurons, leading to intracellular lactate accumulation (in glial cells) and insufficient energy supplement (of neurons).

During cerebral ischemia, lactate is a significant fuel for neurons (100). Otherwise, the intracellular accumulation of lactate overproduced in glial cells can trigger inflammation, oxidative stress, apoptosis, and excitotoxicity (87). In this review, it suggests that the timely utilization of lactate not only benefits the neuronal energy supplement, but also prevents harmful cell response resulted from glial lactate accumulation. MCT1 upregulation in ischemic brain plays a protective role in I/R injury (14, 16). Specifically, lactate is overproduced as a result of enhanced glycolysis, and MCT1 can possibly regulate lactate transport and balance its distribution among neural cells in cerebral ischemia. For one aspect, MCT1 metabolically supports neuronal survival. For another, MCT1 probably alleviates the intracellular accumulation of overproduced lactate and rescues the risk factors of I/R injury (Figure 3). Otherwise, the dysfunction of MCT1 leads to its incapability in the regulation of lactate transport and contributes to the activation of cellular response under I/R injury. Therefore, this review provides a promising preventive strategy for cerebral ischemia—lactate metabolism and MCT1.

## Author Contributions

MZ drafted the manuscript. YYW and YB assisted with the revise of manuscript. LMD assisted with the draft the manuscript. HG critically revised the manuscript. All authors contributed to the article and approved the submitted version.

## Funding

This work was supported by grants from the National Natural Science Foundation of China (Grant No. 82171840) and Natural Science Foundation of Chongqing (Grant No. cstc2021jcyj-msxmX0281).

## Conflict of Interest

The authors declare that the research was conducted in the absence of any commercial or financial relationships that could be construed as a potential conflict of interest.

## Publisher's Note

All claims expressed in this article are solely those of the authors and do not necessarily represent those of their affiliated organizations, or those of the publisher, the editors and the reviewers. Any product that may be evaluated in this article, or claim that may be made by its manufacturer, is not guaranteed or endorsed by the publisher.

## Abbreviations

BBB: blood brain barrier
CD147: cluster of differentiation 147
CNS: central nervous system
GSH: glutathione
IL23/IL17: interleukin 23/interleukin 17
I/R: ischemia/reperfusion
MCT: monocarboxylate transporter
MCTs: monocarboxylate transporters
mPFC: medial prefrontal cortex
NAD+: nicotinamide adenine dinucleotide
NADH: nicotinamide adenine dinucleotide
NADPH: methylenetetrahydrofolate reductase
NMDARs: receptors of the N-methyl-D-aspartate type
OL: oligodendrocyte
OLs: oligodendrocytes
TCA: tricarboxylic acid
TLR2/TLR4: toll-like receptor 2/toll-like receptor 4
TMDs: transmembrane domains.
